# The Poor Man's Home.—IX

**Published:** 1897-10-16

**Authors:** 


### Modern Sociology.

### THE POOR MAN'S HOME.—IX.

### The Municipal Landlord.

"Glasgow a Model Municipality" was the title of an article in one of the reviews some time ago, and the phrase was well-deserved. It is thirty years since Glasgow as a city took up the task of clearing out its slums and building healthy houses in their place; and though other towns have taken up the work and may even have improved on its example, Glasgow must be regarded as a pioneer in the evolution of the municipal conscience. When the Glasgow City Improvement Trust commenced its work the ratepayers grumbled—as ratepayers are wont to do—at the tax (sixpence in the pound of rental) imposed to meet the expense of the first operations. To-day no one would deny that the benefit to the city as a whole was worth the cost of the experiment, although that cost amounts to-day to nearly £600,000. The first intention of the city was by no means to become such a large holder of house property as it now is. Its first acts were to demolish slum property, widen some existing streets, make a few new ones, and lay out a public park. But when this was done the Corporation of Glasgow found itself the proprietor of much land in and about its new streets which, as it appeared, no one was willing to build on. Moreover, in the course of their investigations the Trust found that the houses of the poor were such as to make them the hives and breeding-places of immorality and often of crime. These conditions they set themselves to remedy by providing houses for all classes of the poor, from the industrious artisan to the casual and unskilled labourer.

The original intention was to build dwellings for those who can't strictly be called the poor, and the Trust has been blamed for going beyond its proper sphere and building houses with rents which, as an American observer notes, the lowest classes of the poor cannot afford to pay. "The tenants of the Saltmarket model dwellings to-day," says Dr. Gould, "are, in the main, artisans in receipt of good wages. Some of them are shopkeepers and clerks, and even a few families are found apparently so well off that they can afford to hire servants."

The excuse for this is that many of the sites which the Improvement Trust found itself the proprietor of were in places which the very poor did not frequent; if they were to be occupied at all it must be by the richest of the working class. The Corporation is thus at the present time the owner of dwellings of four and five apartments, and even of some rather handsome shops. The Trust has, however, built also houses for the really poor, the people who never expect to call more than one or two rooms their own. The authorities were at first unwilling to build any of those one-room dwellings which have so often been made the reproach of Glasgow, but it was found that many families could not afford to pay the rents which, if they were to be anything but a loss to the community, it was necessary to charge for larger dwellings. The single-apartment houses are, however, very large, and can be easily divided into almost separate rooms, so that from the point of view of health they are not very objectionable; but, on moral grounds, their existence is to be deprecated. The one-room tenements rent at 3s. 3d. per week, the rent including the water rate, but no other taxes. In Scotch tenements generally the taxes are paid by the tenant, and allowance for this, at the rate of about 5s. per room per annum, must be made in comparing rents there with those in England. Each tenement has a cooking range, and a "bunker" which holds about 300 lbs. of coal. In some of the blocks there are dust-shoots, but these have proved unsatisfactory in many cases, tenants throwing into them all manner of solid refuse. A dust-bin, which is cleaned every day, is now more general. There is a laundry for each nine families. This seems insufficient; one for every six families is a proper proportion, as people in the class who occupy these dwellings generally wash once a week. Nevertheless, there is no difficulty in getting tenants, and these say that the Corporation houses are about 15 per cent. cheaper than those of private landlords. For two-room houses 4s. a week is charged, and for those of three rooms 5s. 9d. In some of the blocks there is a resident caretaker; these are much the best kept. In others, the superintendent stays elsewhere, and merely visits the blocks at intervals; but this plan seems very ineffective in securing cleanliness. The sanitary arrangements in them are, however, good, and well looked after, and these blocks have been very free from infectious disease. The tenants are of a respectable class—indeed, some are too well off for the dwellings they occupy. Dr. Gould, the American commissioner, from whom we have quoted before, notes as a curious and regrettable characteristic of Glasgow that many people take a small and dirty lodging, even when they are able to afford a better, in order to save a sixpence, while they are ready to spend far more on whisky, tobacco, or any other luxuries. It cannot be said, however, that English people of the same class are free from this fault. At least, Lord Monkswell mentions, in the *North American Review* the case of a family in London whose united earnings amounted to £7 a week, yet they all—father, mother, two grown-up sons, and as many daughters—huddled together in one room, preferring to spend their money on visits to theatres and music halls, varied by frequent Saturday excursions, week-ends at Brighton, and other pleasures.

Some admirable tenements for artisans have been put up by the municipality of Liverpool. The site which the Corporation took over was formerly occupied by houses of the worst class, in which people were huddled together with most unhealthy closeness. The average was 282 per acre. As in Glasgow, the Corporation did not wish to undertake building, but the land having been offered at auction and finding no purchaser, something had to be done with it. Therefore they have built the Victoria Square artisans' dwellings. These contain 21 one-room tenements, 164 of two rooms, and 86 of three. There is also a house of four rooms for the superintendent, and 12 shops. The one-room houses are 12 ft. square. In those of two rooms the living-room measures 13 ft. by 12 ft. 4 in., and the bed-room 15 ft. 3 in. by 9 ft. 4 in. It seems a pity that the one-room dwellings are not larger if they are to be built at all, since they must be both living and sleeping rooms for their occupants.

